# Relationship between smoking status and tooth loss: Findings from national databases in Japan

**DOI:** 10.2188/jea.17.125

**Published:** 2007-07-18

**Authors:** Takashi Hanioka, Miki Ojima, Keiko Tanaka, Hitoshi Aoyama

**Affiliations:** 1 Department of Preventive and Public Health Dentistry, Fukuoka Dental College, Fukuoka, Japan.; 2 Department of Preventive Dentistry, Graduate School of Dentistry, Osaka University, Osaka, Japan.; 3 Department of Public Health, Faculty of Medicine, Fukuoka University, Fukuoka, Japan.; 4 Tochigi Prefectural Medical and Social Welfare College, Utsunomiya, Tochigi, Japan.

**Keywords:** Smoking, Tooth Loss, Health Care Survey, Nutrition Survey, Dose-Response Relationship, Drug

## Abstract

**Background:**

A causal association between cigarette smoking and periodontal disease has been established. The present study examined the association between smoking and tooth loss using national databases in Japan.

**Methods:**

Records of the Survey of Dental Diseases and the National Nutrition Survey in 1999 were linked electronically using common identification. Records of 3,999 subjects aged older than 40 years were analyzed using logistic regression models, controlling for confounding factors, such as age, frequency of tooth brushing, body mass index, alcohol consumption, and intakes of vitamin C and E.

**Results:**

Prevalence of tooth loss in terms of having less than 19 existing teeth was 37.3% overall. Smoking rates differed in males (45.6%) and females (7.8%). The prevalence of tooth loss in nonsmokers, former, and current smokers was 28.5%, 38.6%, and 36.9% in males, and 38.6%, 34.3% and 38.9% in females, respectively. Adjusted means of existing teeth controlling for confounders by smoking status were 21.5, 19.7 and 18.2 in males and 19.0, 19.2 and 16.4 in females, respectively. The association of tooth loss was non-significant in former smokers but significant in current smokers: adjusted odds ratios (95% confidence intervals) relative to nonsmokers in males and females were 1.29 (0.92-1.80) and 0.86 (0.46, 1.60) for former smokers and 2.22 (1.61-3.06) and 2.14 (1.45-3.15) for current smokers, respectively. A dose-response relationship between lifetime exposure and tooth loss was seen (P for trend <0.0001).

**Conclusion:**

The findings of this cross-sectional study of a nationwide population of Japanese indicated an association between smoking and tooth loss.

Cigarette smoking is associated with periodontal disease. A causal association has been established according to the guidelines for strength of evidence.^1^ With respect to dental caries, a causal association with smoking was suggested for caries on the root surface of teeth. Periodontal disease would increase the possibility of caries development in terms of exposing the root surface to acid-producing bacteria due to recession of the gingival margin.^1^ Periodontal disease was the predominant reason for tooth extraction in persons aged over 45 years in a nationwide survey conducted in Japan.^2^ Therefore, Japanese smokers may lose more teeth than nonsmokers due to smoking.

Because various life-events may influence tooth extraction, confounding factors should be considered when examining the association between smoking and tooth loss via periodontal disease caused by smoking. Nutrients and foods, in particular, vitamin C^3^ and E^4^ intakes, body mass index (BMI),^5^ and alcohol consumption^6^ may be associated with periodontal disease. Behavioral factors such as oral-health practices^7^ and regular dental visits^8^ for the prevention of dental diseases may influence the retention of teeth. Socio-economic status (SES) has also been used to examine the association .^9^

In the United States, an association between smoking and tooth loss has been identified.^6^^,^^10^^-^^16^ Such an association was also found in subjects residing in Sweden,^17^^-^^19^ Australia,^20^ Iceland,^21^ Jordan,^22^ Brazil,^23^ and Kuwait.^24^ In South-East Asian countries, only a few studies have reported on the association between smoking and tooth loss. These studies were conducted in Japan: Current smoking was significantly associated with tooth loss in workers by controlling for several confounders. The odds ratio (OR) and 95% confidence interval (CI) was 1.53, (1.20, 1.96).^25^ Tooth loss in terms of having less than 19 teeth was significantly associated with smoking in elderly subjects of 74 years of age.^26^ A positive association between smoking and experience of tooth extraction was identified in pregnant women.^27^ These findings suggest an association between smoking and tooth loss in Japanese.

The aim of the present study was to examine the association between smoking and tooth loss at the national level using national databases.

## METHODS

### Study Population

National aspects of the dental disease status in Japan have been assessed via reports of the Survey of Dental Diseases (SDD); however, the survey did not include information on the smoking status. The target population was based on the principal extract of a nationally representative sample, and the same population was assessed in the National Nutrition Survey (NNS), which included information on the smoking status. The NNS has been conducted annually since 1945 to monitor health conditions and dietary intake.^28^ The study population was derived from 300 of 1,000 areas, which were selected by employing a two-stage cluster sampling method in the Comprehensive Survey of the Living Conditions of the People on Health and Welfare. The SDD, which involved the same sample population as that of the NNS, has been conducted every six years since 1957. Data of the NNS and SDD were stored independently. We obtained the databases from the Ministry of Health, Labour and Welfare with permission for analytical use and linked the records based on the identification number of each household, age, and sex. Records of 12,763 and 6,903 subjects in the NNS and SDD, respectively, aged 1 year and older in 1999 were evaluated; as a result, 6,805 records were linked successfully. Because only 6 subjects had less than 19 teeth in the age group of younger than 39 years, 2,806 records of subjects aged younger than 39 were excluded for analyses. Finally, 3,999 records of subjects aged 40 years or older were analyzed.

### Measurements

According to the description of the NNS, dietitians visited eachhousehold to collect information regarding the dietary status. Among numerous variables of the NNS, several variables were selected a priori as possible confounders prior to the application of national databases. Variables regarding smoking status, sex, age, BMI, status of alcohol consumption, and intakes of vitamin C and E were analyzed. Smoking status was defined in the questionnaires as: "current smoker", an individual who currently smokes and has smoked more than 100 cigarettes in total after starting smoking; "former smoker", an individual who has previously smoked more than 100 cigarettes in total after starting smoking but does not currently smoke; and "nonsmoker", an individual who has never smoked or smoked no more than 100 cigarettes in total after starting smoking. Subjects were partitioned into four age groups: 40-49, 50-59, 60-69, and 70+ years. The status of alcohol consumption was divided into three groups: current and former drinkers and subjects who have never drank. Habitual drinking was defined in the questionnaires as an intake of more than about 20 g of ethanol per day for 3 days or more per week. The amount of alcohol consumption was derived from responses regarding the type of alcoholic beverages. Based on the Recommended Dietary Allowances and Dietary Reference Intakes for Japanese (6th Revision), vitamin C and E intakes were categorized into two groups according to reference values of the recommended dietary allowance.^29^

According to the description in the report of the SDD, participants visited designated locations where calibrated dentists examined and recorded the status of each tooth which was present in the oral cavity regardless of the degree of eruption. The number of existing teeth was derived from the summation of sound, filled, and decayed (untreated) teeth in the SDD. Lost teeth were defined as permanent teeth (excluding third molars) lost due to extraction or dropout. Dental implants were included for lost teeth. Participants were interviewed for the frequency of tooth brushing. Variables regarding dental visits and the SES were not available in these databases.

### Statistical Methods

Due to the national objective of the "8020 Movement", a program promoting the retention of 20 teeth till 80 years of age in Japan, a category of 0-19 or more than 20 teeth has functioned as a marker of tooth loss in Japanese.^26^ The association between smoking and tooth loss in terms of having less than 19 existing teeth, i.e., subjects who did not meet the national objective, was analyzed using logistic regression in males and females. In the logistic regression model, unadjusted ORs of current and former smokers relative to nonsmokers were analyzed. Then, adjusted ORs were calculated in the multiple logistic regression model by controlling for confounders (reference category): age (40-49 years), frequency of toothbrushing (more than twice a day), BMI (<25.0 kg/m^2^), status of alcohol consumption (never), daily intakes of vitamin C (100+ mg) and E (10+ mg for males and 8+ mg for females).

The dose-response relationship was evaluated using life-time exposure, with the Brinkman Index, which was calculated by daily consumption multiplied by years of smoking, as the exposure dose. Records of former smokers were excluded for analysis because the effect of smoking may be diluted after quitting smoking, and the dilution effect, in general, depends on the time after stopping smoking. This variable was not available. Trend analysis was performed by entering categories of life-time exposure as continuous variables. These analyses were conducted with statistical software (SPSS^®^ 13.0J, SPSS Inc., Chicago, IL). The level of significance was set at 5%.

## RESULTS

Among 3,999 subjects analyzed in the present study, 59.5% were female, and 53.1% were 60 years old or older (Table 1). The distribution of nonsmokers, former, and current smokers was 62.6%, 14.4%, and 23.1%, respectively and differed greatly between males and females: 23.2%, 31.2%, and 45.6% in males and 89.3%, 2.9%, and 7.8% in females, respectively. The rate of current smokers decreased with age, while that of former smokers increased.

**Table 1.** Distribution of the smoking status by age group and sex.

Age (years)	Non-smoker	Former smoker	Current smoker	Total
		Male		
40-49	64 (19.9)	69 (21.5)	188 (58.6)	321 (100)
50-59	105 (26.6)	83 (21.1)	206 (52.3)	394 (100)
60-69	121 (23.6)	180 (35.1)	212 (41.3)	513 (100)
70+	86 (22.1)	173 (44.4)	131 (33.6)	390 (100)
Total	376 (23.2)	505 (31.2)	737 (45.6)	1618 (100)

		Female		
40-49	432 (83.6)	16 (3.1)	69 (13.3)	517 (100)
50-59	572 (88.7)	22 (3.4)	51 (7.9)	645 (100)
60-69	615 (91.0)	15 (2.2)	46 (6.8)	676 (100)
70+	507 (93.4)	17(3.1)	19 (3.5)	543 (100)
Total	2126 (89.3)	70 (2.9)	185 (7.8)	2381 (100)

		Total		
40-49	496 (59.2)	85 (10.1)	257 (30.7)	838 (100)
50-59	677 (65.2)	105 (10.1)	257 (24.7)	1039 (100)
60-69	736 (61.9)	195 (16.4)	258 (21.7)	1189 (100)
70+	593 (63.6)	190 (20.4)	150 (16.1)	933 (100)
Total	2502 (62.6)	575 (14.4)	922 (23.1)	3999 (100)

Parcentages in parentheses

Approximately one third of subjects (34.8%) brushed their teeth less than once a day (Table 2). About one fourth was classified with a high BMI score (28.2%) and current drinkers (27.2%). With respect to vitamins C and E, 35.5% and 49.1%, respectively, were classified in lower intake groups. Current smokers were relatively predominant in the following groups: brushing less than once a day (33.2%), current drinking (46.9%), and lower intakes of vitamin C (29.2%) and E (26.7%).

**Table 2.** Number of subjects according to smoking status by confounding variables.

Variables and criteria	Non-smoker	Former smoker	Current smoker	Total
Frequency of daily tooth brushing				
More than twice	1822 (69.9)	325 (12.5)	459 (17.6)	2606 (100)
Less than once	680 (48.8)	250 (17.9)	463 (33.2)	1393 (100)

Body mass index (Kg/m^2^)				
−24.9	1793 (62.5)	393 (13.7)	684 (23.8)	2870 (100)
25.0+	709 (62.6)	182 (16.1)	238 (21.1)	1129 (100)

Status of alcohol consumption				
Never	2172 (80.0)	200 (7.4)	343 (12.6)	2715 (100)
Former	45 (23.0)	82 (41.8)	69 (35.2)	196 (100)
Current	285 (26.2)	293 (26.9)	510 (46.9)	1088 (100)

Intake of vitamin C (mg/day)				
100+	1680 (65.1)	392 (15.2)	507 (19.7)	2579 (100)
<100	822 (57.9)	183 (12.9)	415 (29.2)	1420 (100)

Intake of vitamin E (mg/day)				
10+ (males) or 8+ (females)	1377 (67.6)	261 (12.8)	399 (19.6)	2037 (100)
<10 (males) or <8 (females)	1125 (57.3)	314 (16.0)	523 (26.7)	1962 (100)

Parcentages in parentheses

The percentage of subjects having less than 19 existing teeth was 37.3%, overall (Table 3). The prevalence of tooth loss increased by age, while the rate was similar in males (35.5%) and females (38.5%), and with smoking status as a total; 37.1%, 38.1%, and 37.3% in nonsmokers, former, and current smokers, respectively. Current smokers showed the highest and nonsmokers the lowest rate of tooth loss in each age-group.

**Table 3.** Number of subjects having less than 19 existing teeth according to smoking status by age group and sex.

Age (years)	Non-smoker	Former smoker	Current smoker	Total
		Male		
40-49	2 (3.1)	4 (5.8)	13 (6.9)	19 (5.9)
50-59	14 (13.3)	14 (16.9)	51 (24.8)	79 (20.1)
60-69	35 (28.9)	61 (33.9)	103 (48.6)	199 (38.8)
70+	56 (65.1)	116 (67.1)	105 (80.2)	277 (71.0)
Total	107 (28.5)	195 (38.6)	272 (36.9)	574 (35.5)

		Female		
40-49	26 (6.0)	1 (6.3)	7 (10.1)	34 (6.6)
50-59	111 (19.4)	4 (18.2)	21 (41.2)	136 (21.1)
60-69	280 (45.5)	7 (46.7)	25 (54.3)	312 (46.2)
70+	404 (79.7)	12 (70.6)	19 (100.0)	435 (80.1)
Total	821 (38.6)	24 (34.3)	72 (38.9)	917 (38.5)

		Total		
40-49	28 (5.6)	5 (5.9)	20 (7.8)	53 (6.3)
50-59	125 (18.5)	18 (17.1)	72 (28.0)	215 (20.7)
60-69	315 (42.8)	68 (34.9)	128 (49.6)	511 (43.0)
70+	460 (77.6)	128 (67.4)	124 (82.7)	712 (76.3)
Total	928 (37.1)	219 (38.1)	344 (37.3)	1491 (37.3)

Parcentages in parentheses

Crude ORs and 95% CIs of having less than 19 existing teeth for former and current smokers were significant in males: 1.58 (1.19-2.11) and 1.47 (1.12-1.93), respectively, and non-significant in females: 0.83 (0.50-1.37) and 1.01 (0.74-1.38), respectively (Table 4). The adjusted OR of former smokers by controlling confounders was non-significant in males and females: 1.29 (0.92-1.80) and 0.86 (0.46-1.60), respectively; however, the OR of current smokers was significant in males and females: 2.22 (1.61-3.06) and 2.14 (1.45-3.15), respectively. The adjusted mean of existing teeth was the lowest in current smokers (18.2 teeth in males and 16.4 teeth in females). In males, nonsmokers possessed the highest number of teeth on average (21.5 teeth) and former smokers fell in the middle (19.7 teeth). In females, these figures were similar in nonsmokers (19.0 teeth) and former smokers (19.2 teeth).

**Table 4.** Prevalence, crude and adjusted odds ratios (ORs), and 95% confidence intervals (CIs) of having less than 19 existing teeth and adjusted means of existing teeth by smoking status of of 1,618 males and 2,381 females of older than 40 years of age.

Smoking status	Prevalence (%)	Crude OR (95% CI)	Adjusted OR* (95% CI)	Adjusted means of existing teeth (95% CI)^†^
			Males	
Nonsmoker	28.5 (107/376)	1.00 (reference)	1.00 (reference)	21.5 (20.7-22.3)
Former smoker	38.6 (195/505)	1.58 (1.19-2.11)	1.29 (0.92-1.80)	19.7 (19.0-20.4)
Current smoker	36.9 (272/737)	1.47 (1.12-1.93)	2.22 (1.61-3.06)	18.2 (17.6-18.8)

			Females	
Nonsmoker	38.6 (821/2126)	1.00 (reference)	1.00 (reference)	19.0 (18.7-19.3)
Former smoker	34.3 (24/70)	0.83 (0.50-1.37)	0.86 (0.46-1.60)	19.2 (17.5-21.0)
Current smoker	38.9 (72/185)	1.01 (0.74-1.38)	2.14 (1.45-3.15)	16.4 (15.2-17.5)

* : Based on multiple logistic regression controlling for confounders: age, frequency of daily tooth brushing, body mass index, status of alcohol consumption, and intakes of vitamin C and E. Criteria and distribution of the variables are shown in Tables 1 and 2.

† : Analysis of covariance was employed to calculate adjusted means of existing teeth controlling for the confounders.

The dose-response relationship between smoking and tooth loss was examined in nonsmokers and current smokers (Table 5). The prevalence of tooth loss and crude OR were the highest in subjects with the highest score of lifetime exposure in males and females. Adjusted ORs increased by lifetime exposure and were significant in all lifetime exposure categories in males and females. This trend was highly significant (P<0.0001). Adjusted means of existing teeth decreased from 22.1 to 17.0 in males and from 19.0 to 11.8 in females as lifetime exposure increased.

**Table 5.** Prevalence, crude and adjusted odds ratios (ORs), and 95% confidence intervals (CIs) of having less than 19 existing teeth and adjusted means of existing teeth by lifetime exposure, the Brinkman index score, of 1,113 males and 2,311 females of older than 40 years of age. Former smokers (N=575) were excluded due to the possible effect of quiting smoking on risk reduction.

Lifetime exposure*	Prevalence (%)	Crude OR (95% CI)	Adjusted OR^†^ (95% CI)	Adjusted means of existing teeth (95% CI)^‡^
			Males	
0	28.5 (107/376)	1.00 (reference)	1.00 (reference)	22.1 (21.3-22.9)
1-399	28.4 (29/102)	1.00 (0.61-1.62)	1.99 (1.10-3.59)	19.4 (17.9-20.9)
400-1199	36.6 (191/522)	1.45 (1.09-1.93)	2.20 (1.55-3.11)	19.0 (18.4-19.7)
1200+	46.0 (52/113)	2.14 (1.39-3.30)	2.94 (1.77-4.91)	17.0 (15.6-18.4)
		P for trend^§^ <0.0001		

			Females	
0	38.6 (821/2126)	1.00 (reference)	1.00 (reference)	19.0 (18.6-19.3)
1-399	32.5 (38/117)	0.76 (0.51-1.14)	1.74 (1.06-2.87)	16.4 (15.0-17.8)
400-1199	47.5 (29/61)	1.44 (0.86-2.40)	2.30 (1.26-4.21)	16.8 (14.9-18.8)
1200+	71.4 (5/7)	3.97 (0.77-20.5)	14.5 (2.35-89.2)	11.8 ( 6.2-17.5)
		P for trend^§^ <0.0001		

* : Brinkman index score, duration (years) of smoking times daily consumption (cigarettes)

† : Based on multiple logistic regression controlling for age, frequency of daily tooth brushing, body mass index, status of alcohol consumption, and intakes of vitamin C and E. Criteria and distribution of the variables are shown in Tables 1 and 2.

‡ : Analysis of covariance was employed to calculate adjusted mean numbers of existing teeth with allowance for the study variables.

§ : P for a trend across two categories using multiple regression analysis controlling for the study variables. Four categories of lifetime exposure were entered as continuous variables.

## DISCUSSION

A significant association between current smoking and tooth loss was demonstrated in the survey through Japan. Furthermore, the dose-response relationship between smoking and tooth loss was distinct. Few studies have addressed the dose-response relationship in terms of tooth loss.^11^ Because this study was cross-sectional, a causal association should not be estimated; however, the adjusted OR of former male smokers fell between those of non-smokers and current smokers and the OR in females was less than that of nonsmokers. The association of former smokers relative to nonsmokers was non-significant. These results suggest the benefit of quitting smoking. In a longitudinal study of 789 men in the United States, a period of quitting of longer than 13 years was required for a reduction of the risk of tooth loss to the level of nonsmokers.^30^ Smoking cessation practice is recommended as a part of global public health measures in dental practice.^31^ Because the indicator of tooth loss was derived from the national objective in the present study, the effect of smoking on tooth loss should be strengthened as a measure for the prevention of tooth loss.

The primary mechanism of the effect of smoking on tooth loss, which was addressed previously, is via the effect of periodontal disease. In Japan, the major reason for tooth extraction was periodontal disease (41.8%) and the reason was predominant in subjects over 45 years of age.^2^ In the present study, subjects were limited to individuals of 40 or older years of age. Current smokers may loose teeth via extraction due to periodontal disease rather than dental caries. Cigarette smoking leads to deterioration of the periodontal condition in Japanese adults by analyses of the same databases used in the present study, though the effect would be underestimated due to the low power in detecting periodontal destruction.^32^

Though the study population was derived from national databases, the results in terms of prevalence and distribution may not represent the national status. The source of the surveyed population in the NNS was based on the household.^33^ Only 54% of subjects in the NNS were available for the analysis of tooth loss in the SDD. Subjects who were able to visit for dental examination may be limited and biased. For example, a more health conscious group may have undergone examination. However, smoking rates by sex and age were similar between databases (data not shown). Though the sample may not represent the national average, an association between smoking and tooth loss was demonstrated by the survey which was conducted through Japan.

Discrepancies in the association of former smokers and tooth loss in males, and current smokers and tooth loss in females were apparent between the results from bivariate and multivariate analyses. By bivariate analyses, the association between former male smokers and tooth loss was significant and that between current female smokers and tooth loss was non-significant. The prevalence of tooth loss was similar between nonsmokers and former smokers in males, and nonsmokers and current smokers in females. These findings should be interpreted with caution because the distribution of current and former smokers and subjects with tooth loss differed by several confounders, in particular, age. Current smoking was significantly associated with tooth loss and the association between former smokers and tooth loss was non-significant overall, after confounders were adjusted for the multivariate regression model in males and females. Finally, more current smokers had less than 19 teeth compared to nonsmokers. Although the smoking rate was apparently different by sex, the effect of smoking on tooth loss was significant in males and females. Furthermore, the dose-response relationship was also distinct.

Though the smoking rate decreased with age, tooth loss increased in later life. This phenomenon might be contradictory. However, because the effect of smoking, in general, appears in later life, adjustment of age as a confounding variable compensated for the influence of age. The prevalence of tooth loss was almost equal in males and females, while the smoking rate was higher in males than females. In females, other factors which were not entered into the multivariate model may influence the association. For example, females in general prefer sweets, which is one of the major risks of dental caries. However, the evidence is not sufficient to infer a causal association between smoking and coronal dental caries.^1^ The bone mineral content is an important factor for supporting periodontal tissue. Menopause may increase the risk of tooth loss in females via periodontitis due to the effects of osteoporosis; estrogen replacement therapy protects against tooth loss and reduces the risk of total tooth loss.^34^ The effect of smoking on coronal dental caries is controversial. The effect on caries in the crown of the tooth, if any, would appear in younger subjects than those examined in the present study, because premature teeth are susceptible to coronal dental caries.

Tooth loss typically occurs by means of dental extraction; thus, it is possible that smokers were more likely to seek dental care than non-smokers due to oral problems. Indeed, a variety of oral symptoms and diseases are associated with smoking. Though data pertaining to access to oral health care were not available in the present study, smoking was significantly associated with tooth loss in young Japanese women upon consideration of the family income and education.^27^ SES is often cited as a potential confounder; however, it may not have an independent effect but affect disease risk through its association with smoking. Selfrated health may be associated with social inequality in Japan.^35^ However, the negative association of smoking with SES is controversial: Current smokers were likely to be more educated among men and women.^36^ In contrast, relationships between the per capita income, unemployment rate, and current smoking were demonstrated.^37^ The effect of SES may have been weak at the time when these surveys were conducted in 1999, because the smoking rate of males overall approached half. Some variables used for adjustment, for example, frequency of daily toothbrushing and nutritional status, could compensate for the direct effect of SES, if any, on tooth loss.

The strength of the present study corresponded to the outcome derived from a nationwide population. The health consequences of a high smoking rate in terms of tooth loss in Japan warn of a future burden regarding oral health in developing countries where cigarette consumption is increasing.^31^

In conclusion, the findings of this cross-sectional study of a nationwide population in Japan indicated an association of smoking with tooth loss. Further studies should be conducted to examine the causal association using possible confounders including SES and behavioral factors.
